# E-Textiles for Sports and Fitness Sensing: Current State, Challenges, and Future Opportunities

**DOI:** 10.3390/s24041058

**Published:** 2024-02-06

**Authors:** Kai Yang, Stuart A. McErlain-Naylor, Beckie Isaia, Andrew Callaway, Steve Beeby

**Affiliations:** 1 Winchester School of Art, University of Southampton, Southampton SO23 8DL, UK; ky2e09@soton.ac.uk; 2 School of Sport, Exercise and Health Sciences, Loughborough University, Loughborough LE11 3TU, UK; s.a.mcerlain-naylor@lboro.ac.uk; 3 Centre for Flexible Electronics and E-Textiles (C-FLEET), School of Electronics and Computer Science, University of Southampton, Southampton SO17 1BJ, UK; r.j.isaia@soton.ac.uk; 4 Department of Rehabilitation and Sport Sciences, Bournemouth University, Bournemouth BH12 5BB, UK; acallaway@bournemouth.ac.uk

**Keywords:** e-textiles, wearable technology, sensors, sports, fitness, performance, injury, monitoring, rehabilitation

## Abstract

E-textiles have emerged as a fast-growing area in wearable technology for sports and fitness due to the soft and comfortable nature of textile materials and the capability for smart functionality to be integrated into familiar sports clothing. This review paper presents the roles of wearable technologies in sport and fitness in monitoring movement and biosignals used to assess performance, reduce injury risk, and motivate training/exercise. The drivers of research in e-textiles are discussed after reviewing existing non-textile and textile-based commercial wearable products. Different sensing components/materials (e.g., inertial measurement units, electrodes for biosignals, piezoresistive sensors), manufacturing processes, and their applications in sports and fitness published in the literature were reviewed and discussed. Finally, the paper presents the current challenges of e-textiles to achieve practical applications at scale and future perspectives in e-textiles research and development.

## 1. Introduction

Wearable technologies are now accepted and widely used in multiple sports and fitness activities across all levels of performance, from recreational to elite, in individual and team sports, and including non-disabled and disabled athletes alike [1]. Wearables can be used to monitor a wide variety of biosignals (e.g., heart rate and muscle excitation) and can also track performance (e.g., distance and speed) and the technique (e.g., joint angles) used to produce that performance. The most common types of existing wearables are typically wrist-worn smartwatches, chest straps, devices mounted on or in the footwear, or, more recently, those located within sports clothing [2]. Analysis of subsequent data can be used to gauge improvements in fitness, help mitigate injury risk [3,4], inform recovery [5], monitor technique [6], or, at a consumer level, simply provide motivation [7]. The typical sensors used to date are inertial measurement units (IMUs comprising accelerometers, gyroscopes, and magnetometers), Global Positioning Systems (GPS), and heart rate (electrocardiography, ECG) and muscle excitation (electromyography, EMG) sensors. 

Electronics textiles (e-textiles or smart fabrics) are advanced textiles that include electronic functionality ranging from conductive tracks to sensing/actuating, communications, and microprocessing [8]. The global market for e-textiles is projected to reach around $15 billion by 2028 [9]. E-textiles are a platform technology for wearables that are highly relevant to sports and fitness applications. The motivation for incorporating sensing and electronic functionality into textiles for sports and fitness applications is evidenced by the growing number of clothing-based wearable devices aimed at this sector. Examples include STATSports Apex Athlete series [10] and Catapult One [11], both of which are GPS-based tracking devices also incorporating IMUs, where the electronics containing sensing, communication, and processing capabilities are implemented in a conventional rigid form with the modules located in a pocket on the garment. GPS-enabled smart watches or footwear could provide similar GPS data; however, their use is not permitted in most sports where physical contact between participants is possible, but this is dependent on the changing rules of each sport.

**Figure 1:**
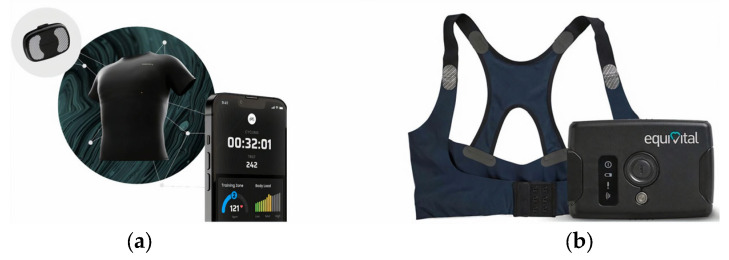
(**a**) Prevayl ECG vest [15], reproduced with permission from Prevayl; (**b**) Equivital multi-parameter monitoring system [16], reproduced with permission from Equivital.

Textiles provide a comfortable, ubiquitous platform that individuals are entirely familiar with. Considerable effort has gone into engineering technical textiles for sportswear [12], where the market is dominated by household brands such as Nike, Adidas, and Puma. E-textiles technology offers the ability to further enhance sportswear functionality by invisibly integrating sensors, microprocessors, and communications into garments [13,14]. This approach can potentially improve compliance with technology amongst users, develop the ecological validity of the data where sensing can happen in the natural sporting environment, and collect data on more important metrics about populations remotely to develop sensing algorithms and interventions. However, at present, the level of integration of the electronic functionality within the garment is typically limited to separate modules that fit into a pocket located on the clothing, as illustrated by the STATSports and Catapult cases. A few other examples do include additional functionality in the textile. Prevayl’s Smartwear incorporates conductive textile electrodes connected to the electronic unit with a 512 Hz sampling frequency to detect the ECG signal and display the information on a smartphone (Figure 1a) [15]. The product is used for both amateur and professional athletes. Equivital LifeMonitor with built-in multi-sensors can monitor ECG, respiration, tri-axis accelerometry, and temperature (Figure 1b). The product is mainly used in training (e.g., military) and health and safety monitoring in harsh working environments (e.g., fire-fighting), but it can also be used in professional sports monitoring (e.g., car races, bike races) [16]. Despite the advances in e-textiles, it is not straightforward to achieve the required reliable and robust electronic and sensing capability in textiles in a manner that has minimal effect on the properties of the fabric. Most existing e-textile technology does not yet deliver practical solutions that replicate the levels of sensing, processing, and communication functionality achieved with the separate, rigid, discrete modules located in a pocket within the garment. 

This review contains a comprehensive evaluation of the capability of existing technologies and approaches to addressing a wide range of sports/fitness-related applications and looks forward to the development of e-textiles and the corresponding research challenges. Section 2 introduces the role of technology in sports in general, reviews the measurements and parameters that are of interest to the participant or coaching/support team, and highlights a use-case scenario of monitoring training loads in an attempt to promote beneficial adaptations and reduce injury risk. Section 3 discusses the commercial non-textile and textile-based wearable devices in sports and fitness applications. Section 4 outlines the drivers for research in e-textiles and presents research-based examples of existing e-textile systems using different sensing technologies. Section 5 discusses future opportunities and the associated research and development required to realise practical e-textile solutions that assist individuals across all abilities and age ranges.

## 2. Technology and Sensing in Sports and Fitness

### 2.1. Current State and Future Considerations of Technology in Sports Science

Technology has always played an important role in sports science; without it, much of the scientific foundations within its sub-disciples would not have been possible. Historically, sports science was often restricted to a laboratory-based setting, in subject discipline isolation, but the rapid development of technology (sensors, processing, and communications) has allowed the movement from the laboratory to training and competition venues and environments. Calls to enhance the interdisciplinary nature of sports science work [17,18,19,20,21] have slowly been realised in research [22,23,24,25,26], but the call is ever-present. This multidisciplinary approach has happened more rapidly in practice in tandem with the professionalism of multidisciplinary teams [27] involving coaches, strength and conditioning specialists, medical doctors, rehabilitation therapists, physiologists, psychologists, nutritionists, and many others. This has likely happened with the commercial development of technology (e.g., cameras, heart rate monitoring, mobile force platforms), allowing the capture of a variety of data to measure athletic performance and facilitate the design of interventions to enhance performance, recover from injury, and monitor wellness, sleep, and diet. 

Athletes wear smartwatches, fitness trackers, heart rate monitors, and other sensors to track their performance, monitor their health, and analyse their training. Previously outlawed in many competitions, there is a slow but welcome allowance to wear sensing technology in competitions, although sport-dependent. These devices can provide real-time feedback metrics such as heart rate, calories burned, and distance covered, helping athletes optimise their workouts and avoid overtraining. They can also help coaches track the progress of athletes they work with and make informed decisions about training and competition. 

As wearable devices become more advanced, for example, with the development of communication to transfer more information more rapidly and with the development of mathematical processing from inertial sensors, they will provide ever more detailed and accurate data, giving athletes and coaches a deeper understanding of physical and tactical performance, and training loads. Analytics of this data will continue to improve, allowing analysts to identify more complex patterns and trends and make more accurate predictions about athlete performance and injury risk. However, many these processing capacities will lie with large companies interrogating ever-growing data sets. 

Virtual and augmented reality are a growing trend in sports [28,29,30] and have the potential to have a large impact on how we learn skills [31] as they become even more realistic, providing athletes with an immersive training experience that closely mimics the real thing. However, as technology becomes more integral to sports performance, there is a risk that it could create a divide between well-funded teams and athletes and those who cannot afford the latest equipment and technology. This could widen the gap between elite athletes and those who are just starting out, making it even harder for newcomers to break into the sport [32,33,34]. At the same time, there is an opportunity to develop interventions using virtual reality to develop the talent pool of athletes available, as long as practitioners are sufficiently trained in how to use and adapt said interventions. 

From an athlete/coach/practitioner perspective, we already have a deluge of data, often with more metrics and data recorded than can be actioned. Some simple but key questions need to be asked as we progress with these technological developments. Are the metrics measured valid and reliable across a range of populations? As we move from laboratory to different training environments, how rigorous does the setup need to be? As technology develops different metrics, how sensitive are these to change (e.g., minimum clinically or practically significant change or minimum detectable change), and what does this mean for different populations? Are metrics named for trademark and marketing terms obscuring good science or confusing users? Do the users understand what this metric is and how it can be modified through intervention—is it useful? Future academic work will need to focus on developing actionable interventions through knowledge transfer with companies and teams to develop an understanding of training interventions. With a global pandemic of physical inactivity leading to early mortality and mental health conditions [35], there is an opportunity, and a need, to develop technology to enhance our ability to personalize health, rehabilitation, and well-being, which will start to be intervention-led in conjunction with the progression of technology.

### 2.2. Technology for Training Load Monitoring in Sport

A common application of technology within sports is for training load monitoring. Whether physical activity is performed for health and/or social benefits, sporting performance, injury risk reduction, and/or post-injury rehabilitation, a primary concern is the prescription and monitoring of an appropriate ‘dose’ of activity. This is often described as the ‘training load’, representing the demands of the particular activity for the particular individual in the particular context that it was performed [36]. At a simplistic level of understanding, there is hypothesised to exist a ‘sweet region’ of ideal training load, below which prior adaptations may be lost (i.e., disuse) and above which the negative consequences (e.g., tissue damage) of activity may exceed any beneficial adaptations (i.e., overuse) [37,38]. Additionally, the balance between training stimulus and inter-session recovery feeds into a ‘fitness-fatigue’ model of performance [39]. These simplistic models can be applied to various biological systems and their related performance and/or injury effects [40].

Training load is often divided into ‘external’ and ‘internal’ load. External load refers to the activity and work completed (e.g., distance travelled, mass lifted, number of repetitions), whereas internal load refers to the effects of that activity on biological systems [41,42,43]. In this context, ‘load’ may not necessarily refer to the mechanical force experienced but rather a description of the demands of the activity, just as in familiar terms such as ‘workload’, ‘cognitive load’, or ‘viral load’ [44,45]. Each of these categories can be further subdivided into physiological or biomechanical loads. For example, external physiological load may refer to metabolic power, whereas internal physiological load could be the oxygen uptake or cardiovascular demand [46]. These metrics are typically measured using laboratory equipment where a participant would participate in a VO2max (or equivalent) test to measure oxygen uptake or take lactate samples from blood to calculate fatigue levels. Through experimentation, regression equations [47,48] have been created to allow estimates of VO2max, which can then be calculated using input from worn sensors (e.g., heart rate), yet lactate has only recently become non-invasive [49] showing potential for e-textiles in the future.

External biomechanical load may refer to ground reaction forces or centre of mass accelerations, whereas internal biomechanical load may refer to joint contact forces or muscle-tendon forces [44]. These alternate loads can, therefore, be prescribed, monitored, and altered somewhat independently of each other to achieve the overall aims of a training block or individual session, perhaps while also addressing secondary aims relating to injury risk management [50].

Perhaps the most well-known and commonly used technologies for monitoring training load in sports, as already alluded to within this article, are heart rate monitors and GPS or Global Navigation Satellite Systems (GNSS) technologies. GPS is one sub-section of GNSS, with modern GNSS accessing a greater number of satellites and, therefore, potentially greater precision and reliability [51,52]. Both are typically monitored at the torso, heart rate via a chest strap, and GNSS via a unit positioned between the scapulae in a manufacturer-provided elastic harness. Heart rate data are used to calculate metrics such as peak or mean heart rate or time spent within heart rate zones generally corresponding to the use of different physiological energy pathways (e.g., aerobic/anaerobic energy systems). GNSS, on the other hand, can be used to calculate metrics, such as total distance covered, number of sprints, top speed, work:rest ratios, and the time spent in different speed zones. Such training load metrics can not only be used for the prescription and monitoring of training sessions but can also facilitate manual or automated updating of subsequent session designs based on previous differences between prescribed and achieved training loads [53].

One area of growing focus is the need and current relative inability to measure biomechanical training loads outside of a laboratory [45], especially internal biomechanical training loads. That is, the forces experienced by specific tissues within the body (e.g., muscle, bone, tendon, ligament). This is particularly relevant when seeking to associate training load measures with injury likelihood or inform progressive overloading of the tissue during rehabilitation or injury risk reduction programs. GNSS can describe the activity performed (e.g., a 10 km run at a certain speed), and heart rate can indicate the cardiovascular demands of the activity, but neither can describe the effects of the exercise on the musculoskeletal system. Applications of GNSS are further limited in indoor sports (although Local Positioning Systems can be used [54,55]) and when activity is performed with relatively little translational movement (e.g., holding an isometric squat position in a badminton doubles match). As a result, many companies now incorporate an IMU into the GPS unit, with the accelerometers used to measure cumulative accelerations experienced at the back. These are expressed as modified load vectors such as PlayerLoad^TM^ (Catapult Sports, Melbourne, Australia [56,57]) or Dynamic Stress Load (STATSports, Newry, Northern Ireland [58,59]). As the trunk is the heaviest body segment and is positioned proximally on the body, these accelerations have been proposed to represent whole-body mass centre accelerations and, therefore, relate to the ground reaction forces or impact magnitudes experienced by the athlete (i.e., external biomechanical training load). However, this neglects the influence of other body segment accelerations, considerable post-impact shockwave attenuation inferior to the sensor [60], and the contribution of various frequency components to the overall acceleration signal [58,61]. Even an entirely accurate measure of ground reaction force or mass centre acceleration may not correlate with the internal forces experienced by specific tissues if muscle forces are not accounted for [62]. Nonetheless, overall measures of cumulative torso accelerations have been associated with oxygen uptake and heart rate measures [54,63], distinguished between activities [64], and enabled the monitoring of fatigue [56], among other factors.

IMUs positioned elsewhere on the body offer additional measurement opportunities. IMUs on specific body segments can be used not only to quantify technique but also in conjunction with machine learning or other algorithms used to detect certain activities [65,66,67,68] and perhaps quantify the intensity of these movements. Greater insight can perhaps be gained when data from wearable technologies are used alongside existing models of tissue stress response or as inputs to neuromusculoskeletal models. For example, models of bone remodelling [69,70] have been used to quantify a tibial ‘Bone Stimulus’ metric from tibial accelerometry via IMeasureU’s IMU Step system. While this has shown reliable results in running and soccer-related tasks [71,72], studies have demonstrated an inability of tibial accelerometry to represent loads on the tibia bone [61,73]. Such bone forces are more dependent upon muscle forces than any shockwave resulting from the ground reaction force [61]. Nonetheless, physics-based and/or machine-learning models can be used to predict tibial bone loads from wearable technology data (e.g., a pressure-sensing insole and an IMU positioned on the foot) [74,75]. Similarly, open-source methodologies exist for the use of multiple IMUs as input to whole-body musculoskeletal computer simulation models that could also be used to estimate internal biomechanical loads [76,77]. However, the number and/or size of sensors required will likely need to be reduced before any real-world application for training load monitoring, perhaps predicting unknown segment kinematics via machine learning [78,79,80] or optimal control algorithms (i.e., determining the objective or ‘cost function’ of an individual’s movement via inverse optimal control and using this to predict unknown kinematics during the movement) [81,82,83]. These predictions and the selection of optimal sensor positioning and processing may be augmented through the use of synthetic wearable technology data sets based on real or augmented laboratory motion capture data sets [73,84,85,86,87,88]. Alternatively, it may be possible to identify algorithms to recognize certain activities [64,65,66,67] and then scale tissue-specific load estimates from laboratory-based estimates using an intensity measure derived from a reduced number of sensors [89]. The use of e-textile solutions with greater comfort and reduced conscious athlete awareness may somewhat remove the constraint on the number and position of sensors. Any developed tools for the estimation of tissue-specific internal biomechanical training loads may facilitate the prescription and monitoring of specific loads, as well as the ranking of exercises in terms of specific tissue loading [90,91] or the provision of haptic [92,93] or audio [94,95] biofeedback.

There are a number of important considerations when choosing or developing wearable technology for sports, particularly for biomechanical loads. These include hardware mass, dimensions, fixation, sensor range and sampling frequency, and calibration routines [96]. If the technology is intended to be used for injury-related applications, then it should additionally build upon established causal relationships, be applicable without any laboratory-based inputs, and be informed by specific guidelines such as individual- or population-based normative boundaries, thresholds, or trends [97]. Researchers and manufacturers should not forget the context in which the technology is to be applied and the needs and preferences of the user (e.g., athlete and coach) [98]. Finally, we should assess not only the metric itself but also the consequences of prescribing changes in that metric, especially when the metric being used relies on average relationships rather than a direct measure of physiological and/or biomechanical loads.

As stated in a recent International Society of Biomechanics in Sports career award paper, ‘In the future… training and rehabilitation programmes will use wearable and simple imaging technologies to estimate tissue level biomechanics derived from personalised neuromusculoskeletal modelling in real-time in the real-world. The future is not that far away’ [99]. Developments in e-textile technologies may help to make that future a reality.

## 3. Commercialised Wearable Technologies for Sports

A wide variety of wearable technologies have been developed for or used in elite sports and consumer fitness-related applications. The following are examples of commercial devices, including where they have been explored in the scientific literature.

### 3.1. Non-Textile/Clothing-Based Wearables

Wrist-based wearables are the most popular form of wearable technology for amateur athletes and have been used in numerous studies [100]. There are too many commercial smartwatches available to list here, but examples such as the Apple Watch and Fitbit Charge series provide typical functionality associated with such devices. This includes heart rate monitoring, general activity tracking (steps, distance, energy expenditure, floors climbed, built-in GPS) and recognition and tracking of particular sports/activities (e.g., walking, running, cycling, swimming). A comprehensive list of devices and their applications, together with a summary of performance evaluation, has been presented by Cosoli et al. [101,102]. Most smartwatches incorporate optical heart rate measurement by photoplethysmography (PPG). This approach can be subject to motion artefacts, and the quality of the fit and location on the wrist can also affect measurements, especially when active [103]. Error rates are higher when swimming, where arm movements and the water can also affect the PPG-based sensors [104]. Where arm movement is a key aspect of a particular sport (e.g., baseball pitching or tennis serving), the IMUs within smart watches can obtain sufficient kinematic data to provide real-time feedback on arm-related technique, allowing the player to improve their performance, leading to an improvement in ball speed and the pronation movement in serve [105]. The wearable WHOOP wristband is a wristband specifically focused on fitness and health that tracks an individual’s training, recovery, and sleep [106]. It uses PPG sensors to monitor heart rate, heart rate variability, and sleep, and it uses this data to estimate training magnitude whilst exercising and the rate of recovery. Other specialised wrist-based sensors have been developed for particular sports. For example, the Beast^TM^ wrist-based sensor comprises IMUs with the associated data processing and software, providing information tailored to resistance training [107]. The system provides information on repetition speed and power but was found to be less accurate than linear position sensors when measuring speed, which could be due to the position when worn [108]. Ultimately, the location on the wrist is not ideal for monitoring many parameters and measurements are inferred, leading to higher error rates compared to sensors positioned optimally in terms of performance. For example, straightforward measurements such as step counting can be inaccurate compared with foot-based sensors. 

Another commonly used and well-established class of wearable devices for monitoring users when undertaking physical activity is chest straps with heart rate detection. These detect the electrical signal associated with a heartbeat through the skin, and hence, the electrodes located on the strap must be in contact with the skin. The same technique is used for clinical ECG measurements, although chest straps are limited to 2 electrodes, whereas clinical systems can use over 20. Early research into their efficacy was very positive, demonstrating that consumer sports chest straps achieve 99% accuracy when compared to clinical ECG equipment [109]. Studies exploring the suitability of chest straps to provide data enabling measurement of the RR interval (the time between successive R waves in an ECG signal) found that at high activity levels, the Polar H10 chest strap was superior to a Holter device [110]. The Holter device is commonly used as a reference but was shown to be unsuitable for intense activities and large body movements, whereas the chest strap remained unaffected. 

Wearable inertial sensors can be located on the body using pockets in clothing or by simply strapping the sensor onto, for example, a limb. These started with pedometers recording step count/frequency for daily ambulatory monitoring. The limitation of these is that only total steps for the duration of the recording are shown, and no temporal information is provided for the calculation of the rate of change of steps (i.e., were they running or walking). As technology has progressed, inertial sensor-based devices have helped address this limitation, allowing many sports to obtain various spatiotemporal parameters [111].

The TritonWear (first version) system for monitoring swimmers comprises the Triton Unit (firmware version 1.1.2), a small IMU designed to fit under a swimmer’s cap, and data analysis and presentation software. The system can monitor up to 50 swimmers, providing general motion data such as acceleration and swimming-specific data (e.g., stroke count and stroke efficiency) with reported mean absolute percentage error (MAPE) of 0, 2.4, 7.1 and 4.9% for butterfly, breaststroke, backstroke & freestyle respectively for stroke count data [112].

The Xsens MVN Awinda is a real-time human motion tracker that exploits IMUs placed at multiple locations around the body using simple Velcro straps. The devices are connected with a bespoke wireless protocol that enables synchronised data capture [113]. This system has been used to analyse technique during walking [114,115], running [116,117], cycling [118,119], and more novel activities such as goalkeeper diving [120], cricket bowling [121], change of direction tasks [122], and jumping in handball [123]. A similar array of sensors, with the addition of EMG sensors designed to be stuck to the skin, has been developed by dorsaVi Ltd. (Victoria, Australia) [124]. These sensors can be mounted at the optimum position for the activity type and have been developed for elite sports, medical and workplace applications (e.g., to monitor lifting). The inertial sensors have been validated when placed on the medial tibia of each leg and used to predict the ground reaction forces when running. The system achieved errors in the range of 5.4–6.1% across three participants when compared to a force platform [125]. The dorsaVi technology produces comparable kinematic data to the Xsens Awinda system, indicating that the different mounting methods have little effect on the data collected [126].

### 3.2. Clothing and Textile-Based Wearables

IMUs located within clothing have been developed for a range of specific sports. For example, sensors placed along the spine can be used to identify swimming stroke and phase [127], commercialised with the Incus Nova wearable [128]. This sits in a pocket between the shoulder blades and can also provide data when running. This highlights the broad applicability of IMUs, where the same hardware can provide data for each activity type, subsequently requiring the appropriate analysis. The STATSports Apex Athlete series [10] and Catapult One [11] GPS devices are designed to monitor an athlete’s performance in field sports where parameters such as the distance covered, number of sprints, time in different speed zones, and top speed are monitored. These garment-based wearables allow the electronics to be placed on the upper back between the scapulae, where they are safe and relatively unobtrusive. The STATSports Apex, for example, has been compared favourably with radar-based tracking technologies [129] and has been used to gain insight into the performance of football players in different age groups, the results of which could inform training programs [130]. The Nadi X, developed by startup company Wearable X [131], include accelerometers and haptic feedback in the form of vibrations that are designed to assist with obtaining and maintaining yoga positions. The rigid device mechanically mounts to compression-fitting yoga pants behind the knee.

In the previous examples, the textile itself has no functionality other than to include a pocket or mounting point for the conventional rigid electronic modules. In addition to Prevayl’s Smartwear mentioned in the introduction with ECG electrodes embedded in garments, other manufacturers have also increased the functionality of the textile or garment. Zephyr^TM^ has developed tight or loose-fitting t-shirts and a sports bra that incorporate heart rate and heart rate variability sensing electrodes and a mounting point for a detachable rigid module that monitors breathing and activity via IMUs. The garments are essentially a chest band incorporated within the shirts/bra. The sensors utilise silver conductive fabric electrodes that must be moistened before use, and the module must be removed for washing and recharging [132]. Another smart garment developed by Hexoskin also includes textile electrodes within the close-fitting ProShirt that provide cardiac, respiratory, and activity monitoring [133]. The associated electronics, IMUs and power supply are again provided in a rigid electronic module that sits within a pocket in the garment. The validity and reliability of the Hexoskin smart shirt for measuring heart rate during strenuous physical activity have been evaluated by comparing results with a Polar Team Pro chest strap [134]. During peak activities with multidirectional upper body movements, the Hexoskin provided erroneous data due to motion artefacts in 4 of the nine athletes studied. Another vest-mounted heart rate monitoring top with two textile electrodes is the Equivital Lifemonitor, but this, too, is subject to motion artefacts and is better suited to lower activity levels [135]. These examples highlight motion artefacts as one of the major challenges with wearable ECG/EMG sensors when used in physical activities. Sensoria offers short-sleeved sports tops and bras that use textile electrodes, a snap-on electronics module, and a Smart sock that utilises three textile pressure sensors under the foot to capture cadence, step count and foot landing technique. The sensors are connected to the Sensoria Core, a small, rigid electronics module that snaps into the dock located at the top of the sock. In comparison with a shoe-based inertial sensor and video analysis, the smart sock provided an excellent measure of cadence (ICC 0.91), speed (ICC 0.86), distance (ICC 0.86) and foot strike pattern (ICC 0.91) [136]. Fabric-embedded electrodes are used in the Athos Core compression garment designed to monitor muscle excitation alongside heart and breath rates. The data collected by the Athos system were found to be comparable with a research-grade surface EMG system [137]. A similar product developed by Strive includes an IMU attached to compression-fitting shorts that include electrodes for detecting EMG signals from quadriceps, hamstrings, and gluteal muscles [138]. A study funded by Strive reported a good correlation between muscle and training loads [139]. Dragonfly Golf is a clothing-based all-body motion analysis system that comprises 18 IMUs attached to a full-body base layer garment [140]. The IMUs are housed in rigid modules that fasten to the corresponding connector on the suit, and this enables sensors to be removed for washing and then re-attached. The suit includes a wiring loom that connects the sensors to the central power supply, and a module that collects and wirelessly transmits the data. The hardware is complemented by player and coaching apps that enable the visualisation of the data and detailed analysis of the golfer’s technique. 

E-textiles do not have to be engineered into full garments. For example, smart sensing sleeves that cover the forearm or full arm have been developed. The Komodo AIO Smart Sleeve is a general-purpose wearable device that incorporates a PPG sensor, IMU, battery and Bluetooth Low Energy hardware in a typical rigid module that snaps onto the inside of the sleeve and sits in contact with the skin [141]. The sleeve is available in both short (lower arm only) or long (full arm length) versions, and in active mode, the device monitors steps, distance, heart rate, sleep, and activity intensity. In health mode, the sleeve has an additional wired electrode that connects to the module and is designed to be placed on the chest. This provides one lead, two electrodes, and ECG data when stationary and cannot be used when training. The level of functionality within the textile is low, with the sleeve simply incorporating magnetic clip-on connectors and a single embedded wire to the top connector. Motus has developed a sleeve for monitoring throwing sports such as baseball, American football and cricket, but the functionality is again provided by a rigid module that sits within pockets on a sleeve or band [142]. Myontec offers a suite of garments, including compression shirts, shorts, waist belts and arm and lower-leg sleeves with IMU sensors and EMG electrodes [143]. The shorts provide similar functionality to the Stive system, with EMG sensing electrodes monitoring the quadriceps, hamstrings, and gluteals. This has been used to longitudinally analyse neuromuscular responses to training [144]. The leg sleeve monitors the tibialis, gastrocnemius and soleus muscles, whilst the belt targets the multifidus and erector spinae muscles in the back. The same module housing the IMU, and other EMG monitoring electronics has been used within each garment using a bespoke snap-on rigid connector. 

This review highlights that many of the commercially available wearables, clothing-related or otherwise, rely on IMUs potentially augmented with GPS or heart rate monitoring systems. The hardware and sensor performance offered is similar across all examples, with some variations in the number of sensors and the method/location of attachment. An additional and key distinction is provided in the supporting application software that collects and analyses the data. It is a crowded marketplace, and despite the market size for wearable technologies and the expected growth, successfully monetising the technology and surviving the competition is challenging [145]. 

## 4. Research in E-Textiles for Sports and Fitness

### 4.1. The Motivation for E-Textiles Research on Sports and Fitness

Textile implementations of sensors and electronics have a large range of potential applications in monitoring progress and performance, reducing injury risk and motivating regular exercise and training. To advance the current commercial products and enable the wide adoption of e-textile technologies in sports and fitness, research has been conducted in the areas of new materials and sensor components, manufacturing methods, system integration and user-centred design. The aims of these studies include improving sensing functions (e.g., accuracy, reliability), user comfort (e.g., flexibility, softness), durability (e.g., washing, wearing) and design (e.g., aesthetic appearance, ease to put on and take off). Other research has related to the charging/power, data acquisition/processing/transmission and the interaction with other technologies (e.g., Internet of Things (IoT), Artificial Intelligence (AI)), which are not within the scope of this review but for which information can be found in published papers [8,146,147]. 

The benefits offered by e-textile implementation depend upon the application and the solution, but there are several common advantages:

- Textile/clothing-based solutions provide a comfortable and familiar platform to users. E-textiles would enable unobtrusive and ubiquitous deployment sensors in clothing. Textile-based soft products are safe in contact sports;
- Textiles are versatile materials that can be designed to change their properties to fit the application needs through the optimal combination of textile materials and structures;
- The integration in clothing will improve compliance (users might forget to use conventional technology, but they always remember to get dressed). Ease of use and increased compliance can provide more data to better inform training and reduce the risk of overload;
- The unobtrusive and seamless integration of miniaturised or flexible sensors is less likely to influence the parameters being monitored. This allows close contact between the sensors and the skin, reducing measurement errors caused by the displacement of the rigid/large sensors relative to the underlying anatomy;
- Multiple sensors can be incorporated into a single platform (e.g., an item of clothing) in multiple body positions rather than requiring users to wear several separate devices;
- Integration of sensing within garments enables sensors to be located at the optimal location on the body and to measure a much wider range of signals than possible with, for example, a smartwatch;
- Information or alerts can be provided through the textile, providing real-time feedback to the user in a single platform.

### 4.2. Research on E-Textiles for Sports and Fitness Using Different Sensing Technologies

IMUs, biopotential electrodes and tactile/pressure interfaces are the most commonly used technologies in sports [148]. Main applications include motion measurement, vital signs monitoring and interactive applications. The level of sensor-textile integration varies from simply attaching the sensor to the wearer using a textile (e.g., elastic strap) to integrating the sensor components (e.g., IMU chip, sensing yarn/ink) to form an integral part of the wearable e-textile.

### 4.3. IMUs for Motion Detection and Joint Function Measurement 

**Figure 2:**
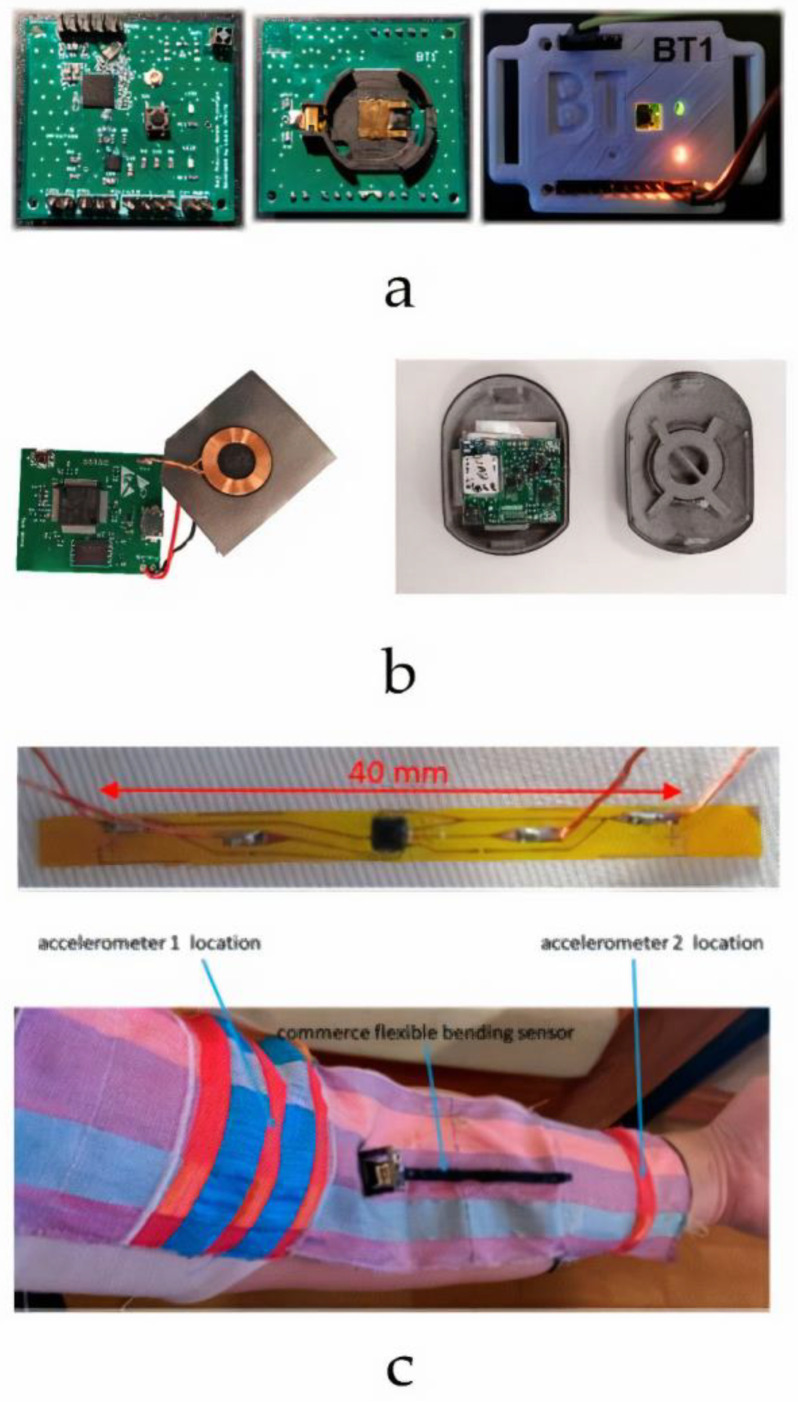
(**a**) The assembled PCB prototypes with a 3D-printed case [152], reproduced with permission from the publisher Elsevier; (**b**) electrical circuit with a protective case [153]; (**c**) IMU filament embedded in a woven textile [154].

The use of IMUs has accelerated with improvements in hardware development and data processing. IMUs have been used in e-textile wearables for sports and fitness due to their low cost, portability, real-time data, and suitability for dynamic movements. They have been used to monitor gestures/poses, range of motion, steps and types of activities (e.g., walking, running, climbing). They can also be used in gait analysis to assess movement patterns and monitor rehabilitation. IMUs have shown moderate to excellent correlations with gold standard approaches for gait spatiotemporal parameters during running [149], but users should be aware of poorer accuracy in high-speed and non-sagittal plane (i.e., abduction/adduction or internal/external rotation rather than flexion/extension) measurements [144,150]. Dahl et al. have validated an IMU system (Opal Gen 2) against a gold standard optical system for sports-related common movements (e.g., cutting, running, jumping) [151]. This study found a good level of agreement between the IMU and the optical system supporting the use of IMUs for sports-related movement/rehabilitation assessment, although the paper stated that continued validation and improvement of sensor accuracy is required. Jenkins et al. have investigated the use of an IMU (LSM6DSLTR) to monitor body configuration in relation to back injury risk in weightlifting exercises [152]. Taborri et al. have used IMU (Invensense ICM 20948) sensors integrated with a support vector machine algorithm to identify faults (e.g., illegal running) during race walking, which has the potential to assist officials in competitions (Figure 2b) [153]. The sensors used in these examples were not directly integrated into textiles, with textiles only used to hold the rigid enclosure of the IMUs in the form of a strap. In order to reduce the size of the sensing system and improve its flexibility, an IMU (MC6470) sensing filament has been developed by soldering the sensor on a narrow flexible circuit (<5 mm wide) and woven into textiles for monitoring joint angles and movement types (e.g., walking, running, climbing stairs) [154]. The flexible circuit was fabricated using photolithography and etching processes described in [13]. Four wires were soldered on the filament to implement the I2C protocol for data, clock, power and ground connections and woven into a textile that can worn on the arm to measure joint angle (Figure 2c). Although sports and fitness applications were not investigated in the paper, the technology is applicable to many sports and fitness applications involving the monitoring of joint movements. In addition to the standard IMU challenge of signal drift, which requires calibration and effective processing, integration of IMUs into a wearable item (e.g., sleeve, t-shirt, leggings) providing close and reliable contact with the skin is required to eliminate/reduce motion artefacts. Although the filament-sensing yarn approach has improved the integration within the textile, the establishment of scalable and cost-effective manufacturing is required to make it commercially variable. 

### 4.4. Electrodes for Heart Rate, Heart Rate Variability, and Muscle Excitation/Strength 

**Figure 3:**
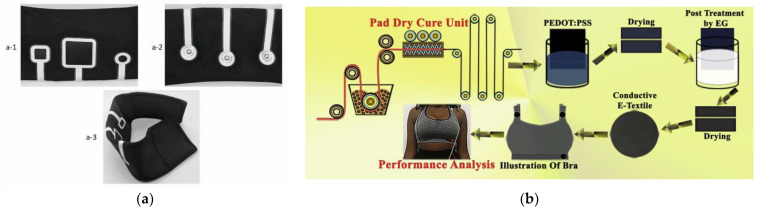
(**a**) ECG bracelet made of printed electrodes with snap button connectors: inner layer (a-1), outer layer (a-2), bracelet form (a-3) [156], reproduced with permission from the publisher Elsevier; (**b**) electrodes made using the pad-dry-cure method and the design of an ECG bra [158], reproduced with permission from the publisher Elsevier.

ECG is the most common biosignal measured for the monitoring of cardiovascular health and to provide early detection of arrhythmias. The conventional Ag/AgCl hydrogel electrodes used in ECG monitoring are not suitable for long-term wearable applications due to the stickiness, moisture evaporation and ease of contamination of the electrodes. Textile-based dry electrodes have been developed for wearable ECG to overcome the disadvantages of hydrogel electrodes (Figure 3). The electrodes form an integral part of the textile, which is then made into a wearable item that provides tight contact between the electrodes and the skin to reduce signal noise generated via the movement of the electrodes. Electrodes are mainly made by depositing conductive inks/paste on textiles using printing [155,156,157], coating the textile in the electrode material solution [158,159] or knitting [160,161], weaving [162,163], or embroidering [164,165] commercially available conductive yarns or bespoke conductive yarns such as graphene-based yarn and PDOT: PSS-modified yarn [166,167]. Sun et al. have developed an ECG t-shirt that can monitor an individual’s heart rate during endurance training [168]. Ousaka et al. demonstrated the feasibility of continuously monitoring ECG during a full marathon to use the technology to prevent sudden cardiac accidents [169]. Bosco et al. investigated the use of a 12-lead ECG strapped to the waist to collect ECG data underwater to analyse heart rate, arrhythmias, conduction abnormalities and ischemic events in relation to various stages in diving [170]. 

EMG is used to monitor muscle excitation using the biological electrical activity signals of the muscle, which can provide useful indicators of muscle activation patterns, fatigue, and performance. EMG has an important role in analysing dynamic movement and understanding the role of a muscle in a specific movement [171]. The measurement of fatigue is important due to its negative impact on cognitive and motor performance, training effectiveness, and risk of injury [172]. The materials and manufacturing process of EMG electrodes are the same/similar to the ECG electrodes described above. Ohiri et al. have incorporated stretchable electrodes and interconnects within a set of athletic compression clothing, including a t-shirt, a pair of shorts, and calf sleeves for large-area EMG monitoring. The modular approach allows the monitoring of the biceps/triceps, quadriceps/hamstrings, and tibialis anterior/gastrocnemius muscles [173]. Liu et al. have developed a wearable EMG with two electrodes embedded in bicycle pants to monitor muscle fatigue during cycling. However, the study found that the EMG signal was affected by artificial noise due to the electrode movement during exercise, leading to lower sensitivity compared to the Ag/AgCl electrodes [174]. 

### 4.5. Piezoresistive, Piezoelectrical, and Capacitive Materials for Pressure, Contact Force/Speed, and Respiratory Rate Measurement

**Figure 4:**
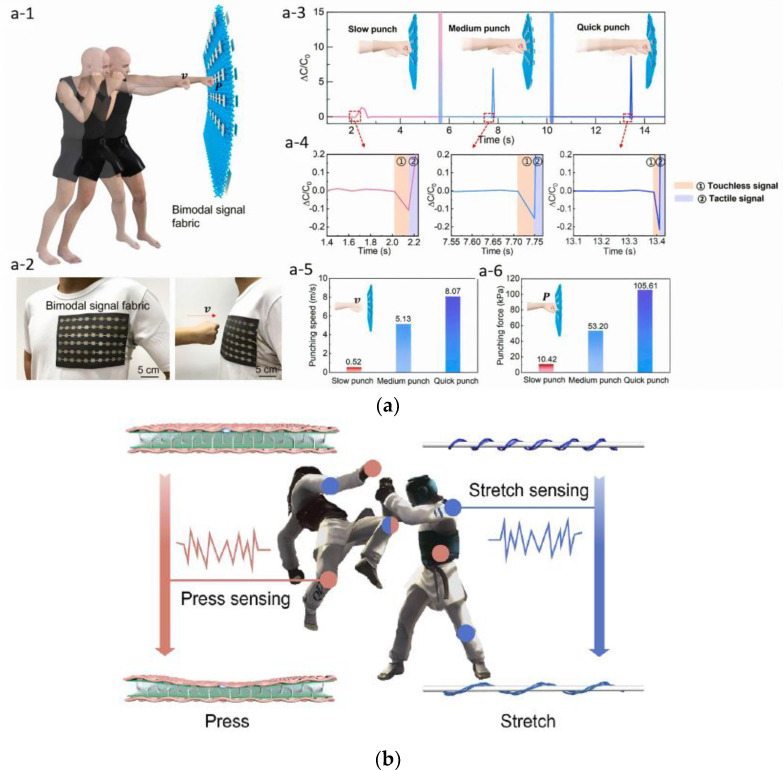
All textile-based sensors to measure (**a**) touchless and tactile signals in boxing: schematic diagram (a-1), a sensor array sewn into a garment (a-2), capacitance response for slow, medium and quick punches (a-3), local amplification diagram response to slow, medium and quick punches (a-4), comparison of punching speed (a-5) and punching force (a-6) [175], reproduced with permission from the publisher Elsevier; (**b**) stretching and pressure in taekwondo [176], reproduced with permission from the publisher Elsevier.

Measuring the pressure/force applied to an object in sports and fitness activities (e.g., running, jumping, boxing) is another important parameter to monitor during training and to assess injury risk, e.g., from collisions. Piezoresistive, capacitive, and piezoelectric materials and devices are commonly used for monitoring pressure and force through the change in electrical resistance or capacitance or the generation of electrical charge in response to pressure or force, respectively. Functional materials are integrated into textiles using standard e-textile fabrication methods (e.g., printing, weaving, knitting, embroidery) to produce conformable and flexible smart garments that can be worn in sports/fitness activities. For example, Ye et al. have developed an all-textile sensing system made of a three-dimensional spacer fabric dielectric layer sandwiched between two nickel-plated plain woven fabric electrodes to detect pressure, distance, and speed and which exhibits good potential for real-time monitoring of human physical combat sports (Figure 4a) [175]. Ma et al. have developed a dual tactile-tension sensing textile made of a three-dimensional spacer fabric sandwiched between two layers of woven textile electrodes (Figure 4b). The textile electrodes are made of silver yarns and core-sheath conductive yarns that can monitor the change of the impendence and capacitance to detect stretching and pressure in taekwondo training [176]. 

These sensors can also be used to monitor the respiratory rate by detecting the expansion and contraction of the chest during inhalation and exhalation, which can be indicated by the change of resistance and capacitance. Sensors can be embedded in chest straps or smart garments to allow real-time monitoring, such as piezoresistive sensors made of silver-plated knitted textiles for respiratory rate monitoring [177]. 

Other applications include temperature-sensing textiles to monitor overheating or hypothermia, moisture-sensing textiles to monitor sweat levels and hydration status, SpO2 sensing textiles to monitor respiratory and cardiovascular health, and electroencephalography (EEG) sensing textiles to monitor cognitive status [147,178].

## 5. Challenges and Future Opportunities 

There are certain technical limitations of e-textile implementations that constrain the practical application and wide adoption of smart fabric technologies. The following challenges need to be considered in the research and development of e-textiles for practical applications:

- The device’s sensing accuracy and the influence of the textile or clothing on the accuracy are challenges in many applications and will require calibration and correct fitting of the wearables. For example, the data gathered from inertial sensors on garments will depend upon how tight fitting the clothing is, as well as potential sensor-to-segment alignment algorithms/procedures. The accuracy can also be affected by sampling rate, sensor drift, and the external environment (e.g., temperature, magnetic interference). Poor ECG/EMG electrode-skin contact will increase the electrode-skin impedance and noise generated by motion artefacts. Accuracy can also be affected by the change in humidity (e.g., sweating), an unstable connection between the electrodes and the electronics, and the degradation of the electrodes. Many piezoresistive materials made of carbon particle-filled polymers exhibit non-monotonic strain response, especially under dynamic conditions due to the cyclical displacement of the conductive network, which can be affected by strain rate, strain history, and maximum applied strain [179];
- The connector used in e-textiles is another component that impacts the size/flexibility and reliability/durability of the sensing system. Sensors must be reliably connected to signal-processing electronics and power sources, and unobtrusively embedding these technologies is not straightforward. Connectors used in e-textiles that allow detachment of the electronic/sensors (e.g., snap fastener, pogo pins, magnetic connector) introduce rigid components with considerable size compared to the sensors, and those providing permanent attachment create potential failure points due to the poor adhesion (e.g., conductive adhesive) and limited capability to accommodate the level of flexing/bending of the textile causing stress on the joints (e.g., soldering);
- Electronic and sensing technologies incorporated during the manufacture of the textile must survive the associated manufacturing process (e.g., weaving, knitting, surface finishing), and e-textile processes must be compatible with mass manufacturing to allow scale-up and reduction of cost. During use, textiles routinely experience physical conditions (e.g., physical wear, bending and flexing, exposure to liquids, and washing) and ensuring solutions are robust and reliable is a significant challenge;
- E-textiles are reliant on conventional primary or secondary batteries, and these are bulky, rigid, and incompatible with the feel of the textile. Although the sensor components are very small (e.g., IMUs) and flexible (e.g., electrodes for biosignals), the presence of the battery has significantly impacted the size and flexibility of the overall system. The integration of the battery with the textile is poor, and the battery will need to be removed prior to washing, which happens regularly because of exercise. The inherently rigid and bulky nature of the battery is intrusive and can spoil the user’s perception of the e-textile technology;
- Co-design with target users to ensure their requirement is considered from the outset of the project. The design needs to consider different requirements associated with sex/gender, age, body size/shape, physical mobility, and digital literacy. Testing with a large user cohort is essential to improve the data quality and explore/address the challenges that may not be noticeable within a small user group;
- E-textiles-based applications that involve data collection, transmission, storage and sharing must consider safety and the implications of data management and associated ethics/privacy issues. The ownership of the data and how it will be used in a safe and ethical way needs to be considered;
- The lack of standards and legislation. The e-textile standards are under development and have not been widely tested by technology/product developers. This has led to the lack of standardised processes and methods, creating challenges in assessing and comparing different work/products. Legislation and incentives are required to address the environmental issues posed by the e-textile;
- Sustainability and circularity need to be considered from the design stage and across the whole product development and product life cycle to minimise the impact on the environment. Products need to be robust to allow longevity. Disassembly needs to be considered to separate different components used in the e-textile for reuse and recycling. In addition to the technology/product developers, other stakeholders (e.g., consumers) need to become involved and accessible infrastructures need to be established to achieve reliable and effective circularity.

Research is required to address the challenges above, which creates opportunities for advancing e-textile technologies and developing fit-for-purpose products. Continuous development of new materials/components and advanced manufacturing processes are required to improve the sensitivity, reliability, durability, and level of integration of textile-based technologies, enabling their long-term use in sports and fitness. Interacting e-textiles with other technologies (e.g., digital technologies, AI) will add new functions for e-textile-based wearable technologies. For example, using AI tools built on the analysis of substantial movement and performance data collected by the e-textile sensors will allow the development of personalised training plans and prediction of performance and rehabilitation progress. Co-design with end users and testing prototypes with a diverse group representing various characteristics of the intended users will allow the researcher to capture valuable insights and feedback to improve the design and increase the likelihood of product market fit. Responsible innovation can only be achieved and implemented with the contribution of different stakeholders involved (e.g., technology developers, manufacturers, distributors, end users, and policymakers), enabling e-textiles to become user-centric, cost-effective, and sustainable solutions.

## 6. Conclusions

Wearable technologies have been widely used in sports and fitness applications at both recreational and elite levels to track performance and health conditions. Wearable sensors provide useful data in vital biosignals and key performance parameters (e.g., speed, distance, acceleration) that can inform training to improve efficacy while reducing the risk of injury. E-textiles provide a platform for the ubiquitous deployment of wearable technologies due to the soft and comfortable nature of the textiles and their suitability for everyday wearing. The level of integration varies among different techniques and applications, from directly inserting the modular sensor/electronic unit (e.g., IMUs, GPS) into a pocket of the garment to having the sensing material truly embedded into the textile to make a sensing textile (e.g., ECG/EMG electrodes, force/pressure sensing fabric) that are connected to a detachable electronic unit. These two types of integration are the most common methods used in commercial e-textile products, and the fully integrated e-textiles system (e.g., electronic units, including batteries) is still in its development stage.

Further advancement is required in order to gain the full benefit of e-textile-based sensors. This includes the development of new materials and components with improved accuracy, reliability and durability; integration of the e-textiles system with miniaturised components to reduce the overall size and its impact on the sports/fitness garment; manufacturing processes that are scalable and cost-effective for both prototyping and volume production; co-design and with end users to ensure product market fit, standardised protocols in data management/privacy and performance testing. In addition, sustainability and circularity need to be considered in each stage of development, as well as the life and post-life cycles of the products. The range of disciplines involved in e-textile research should be broadened to identify new opportunities in sports/fitness applications and address the challenges directly related to the e-textiles and these related to broader disciplines (e.g., energy efficiency, data-informed decisions).

## Data Availability

Data are contained within the article.
